# A randomized controlled trial of digital breast tomosynthesis versus digital mammography in population-based screening in Bergen: interim analysis of performance indicators from the To-Be trial

**DOI:** 10.1007/s00330-018-5690-x

**Published:** 2018-08-29

**Authors:** Hildegunn S. Aase, Åsne S Holen, Kristin Pedersen, Nehmat Houssami, Ingfrid S. Haldorsen, Sofie Sebuødegård, Berit Hanestad, Solveig Hofvind

**Affiliations:** 1 Department of Radiology, Haukeland University Hospital, 5021 Bergen, Norway; 2 Department of Clinical Medicine, University of Bergen, 5020 Bergen, Norway; 3 Cancer Registry of Norway, P.O. 5313, 0304 Majorstuen, Oslo Norway; 4 Sydney School of Public Health, Sydney Medical School, University of Sydney, Camperdown, Australia; 5 Oslo Metropolitan University, Oslo, Norway

**Keywords:** Mammography, Breast cancer, Mass screening, Digital breast tomosynthesis, Randomized controlled trial

## Abstract

**Objectives:**

To describe a randomized controlled trial (RCT) of digital breast tomosynthesis including synthesized two-dimensional mammograms (DBT) versus digital mammography (DM) in a population-based screening program for breast cancer and to compare selected secondary screening outcomes for the two techniques.

**Methods:**

This RCT, performed in Bergen as part of BreastScreen Norway, was approved by the Regional Committees for Medical Health Research Ethics. All screening attendees in Bergen were invited to participate, of which 89% (14,274/15,976) concented during the first year, and were randomized to DBT (*n* = 7155) or DM (*n* = 7119). Secondary screening outcomes were stratified by mammographic density and compared using two-sample *t*-tests, chi-square tests, ANOVA, negative binomial regression and tests of proportions (*z* tests).

**Results:**

Mean reading time was 1 min 11 s for DBT and 41 s for DM (*p* < 0.01). Mean time spent at consensus was 3 min 12 s for DBT and 2 min 12 s for DM (*p* < 0.01), while the rate of cases discussed at consensus was 6.4% and 7.4%, respectively for DBT and DM (*p* = 0.03). The recall rate was 3.0% for DBT and 3.6% for DM (*p* = 0.03). For women with non-dense breasts, recall rate was 2.2% for DBT versus 3.4% for DM (*p* = 0.04). The rate did not differ for women with dense breasts (3.6% for both). Mean glandular dose per examination was 2.96 mGy for DBT and 2.95 mGy for DM (*p* = 0.433).

**Conclusions:**

Interim analysis of a screening RCT showed that DBT took longer to read than DM, but had significantly lower recall rate than DM. We found no differences in radiation dose between the two techniques.

**Key Points:**

*• In this RCT, DBT was associated with longer interpretation time than DM*

*• Recall rates were lower for DBT than for DM*

*• Mean glandular radiation dose did not differ between DBT and DM*

## Introduction

Digital breast tomosynthesis (DBT) in combination with digital mammography (DM) has been claimed to be superior to DM alone in prospective studies of cancer detection in European breast cancer screening programs [1–4]. However, recall rates have been shown to vary between studies.

Globally, a limited number of studies using DBT for screening have reported complete data on interval breast cancers [5–7], and there is presently limited knowledge about the characteristics of the cancers detected with DBT versus DM [5, 7–9]. Further, most studies have evaluated results of DBT in addition to DM, which substantially increases the radiation dose [10–12]. Replacing the DM in DBT + DM with synthetic mammograms (SM), a 2D mammographic image reconstructed from the projection data obtained during the DBT acquisition, has been suggested as a solution and has recently shown promising results with respect to early performance measures in European screening programs [3, 8, 9, 13]. In addition, the sensitivity of DBT among women with dense breasts has been questioned [14–16].

Logistical aspects including increased examination and reading times, the burden on IT systems related to storage, power and speed, and the financial costs are additional aspects that need to be explored to fully evaluate the cost-effectiveness and feasibility of using DBT + SM in organized screening programs.

To address some of the aforementioned gaps in knowledge, we conducted a randomized controlled trial (RCT) using DBT + SM versus DM only: the *To*mosynthesis trial in *Be*rgen (the To-Be trial). The To-Be RCT started in January 2016 and spanned one screening round (2 years). Our study objectives for this paper were to describe the design of this RCT and to report results of interim analyses after the first year of the trial. We compared selected secondary screening outcomes, such as examination time, time spent on screen reading and consensus, rates of cases discussed at consensus, recall rates due to abnormal mammographic findings, and mean glandular dose for DBT + SM (hereafter referred to as DBT) and DM, by mammographic density.

## Material and methods

The To-Be trial is approved by the Regional Committees for Medical and Health Research Ethics and registered at ClinicalTrials.gov (NCT02835625).

### Study design of RCT

**Fig. 1:**
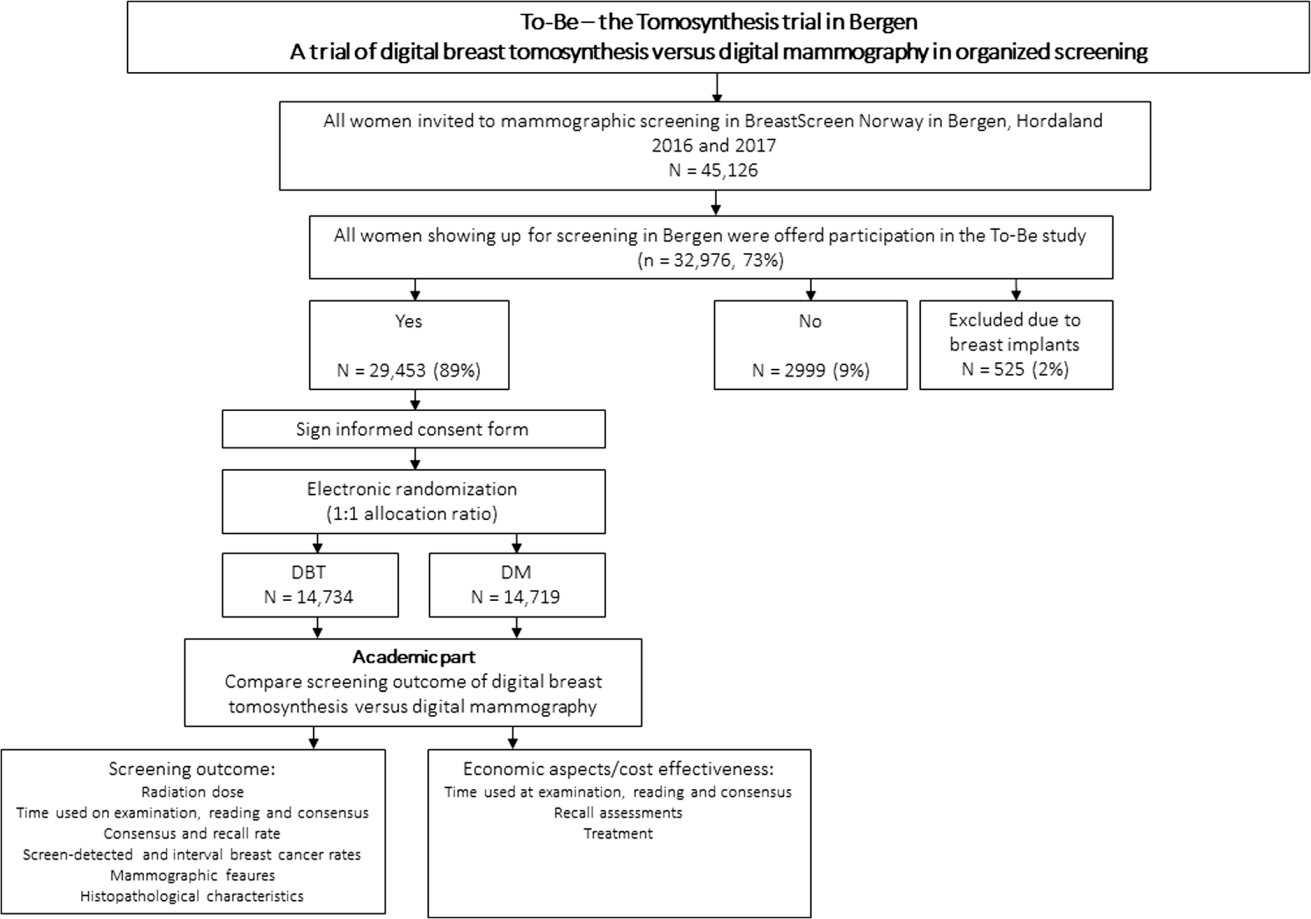
Study design of the To-Be trial in Bergen, a randomized controlled trial using digital breast tomosynthesis in combination with synthesized 2D images (DBT) versus digital mammography (DM), in Breast Screen Norway. Excluded because of a lack of data on mammographic density

The To-Be trial is an RCT aimed at investigating early performance measures and economical aspects when using DBT versus DM in a screening program for breast cancer (Fig. 1). The trial was performed in Bergen, as a part of BreastScreen Norway, a population-based breast cancer screening program targeting women aged 50–69 years. The program is administred by the Cancer Registry of Norway and has been run since 1995. The program is described in detail elsewhere [17].

All women who attended screening at the screening unit in Bergen, 2016 and 2017, received a request about participation in the trial. Those who agreed and signed an individual consent form were randomized to screening with either DBT or DM, using a 1:1 allocation ratio. The target group for the screening site in Bergen counted about 45,000 women for the actual screening round. Assuming an attendance rate of 75% and 90% participation in the trial, the RCT was powered to identify a statistically significant increase of 25–30% in the rate of screen-detected breast cancers. Information related to the screening examination (screening outcome, procedures performed during recall, mammographic features including density, histologic tumor characteristics, treatment etc.) were reported continuously to the Cancer Registry of Norway by the Breast Center at Haukeland University Hospital in Bergen. Participants will be followed for 2 years after screening, to identify interval breast cancers and cancers in the next screening round.

To avoid bias in the performance of the trial, no results of the surveillance or the analyses, except screening attendance rate and participation rate in the trial, were communicated to the professionals who worked in the practical part of the trial.

### Study setting

The To-Be trial was performed in an everyday screening setting. All women underwent standard two-view (craniocaudal and mediolateral oblique views) DBT or DM performed by two radiographers. We used imaging equipment from GE (SenoClaire 3D Breast Tomosynthesis™). The DBT acquisition consisted of nine low-dose exposures over an angle of 25°, reconstructed into 1-mm and 10-mm planes, as well as SM. Screen reading was performed on IDI workstations, each with two 5-megapixel monitors (GE Healthcare MammoWorkstation Version 4.7.0 Image Diagnost). The storage requirement for the raw data and processed image data was 500–3000 MB per examination for DBT and 60–80 MB for DM.

Screening examinations were read using independent double reading. Prior DM screening mammograms were available for subsequently screened women. The standard reading protocol included two views of each breast for DM and two-view synthethic mammograms and 1-mm planes of each breast for DBT. Slabs were vailable for DBT and used in challenging cases, mainly during the consensus meetings. Each breast was assigned a score of 1–5 by each radiologist. A score of 1 indicated screening examination negative for abnormality; 2, probably benign; 3, intermediate suspicion; 4, probably malignant; and 5, high suspicion of malignancy. If either radiologist assigned a score of 2 or higher to one or both breasts, a consensus meeting (hereafter referred to as consensus) with two or more radiologists was held to determine whether to call the woman back for further assessment (recall).

Up to four prior examinations were available at the workstation both for initial screen reading and consensus. Assessment of recalled women included additional mammographic imaging and/or ultrasound, potentially a needle biopsy and sometimes an MRI. Recall assessment took place at the Breast Center at Haukeland University Hospital.

Eight radiologists with 0–19 years of experience in screen film and/or digital mammography (mean 7 years) took part in screen reading, consensus and follow-up assessments (Appendix, Table 5). All radiologists who did screen reading also performed the assessments for recalled women and diagnostic examinations. DBT was available as a diagnostic method at the Breast Center for about 1 year prior to starting the trial, but had not been used for screening. All radiologists attended a training session with DBT before they started screen reading in the trial. Moreover, a pilot study performed 8 weeks pre-trial included about 300 DBT screening cases.

### Study population of interim analyses

**Fig. 2:**
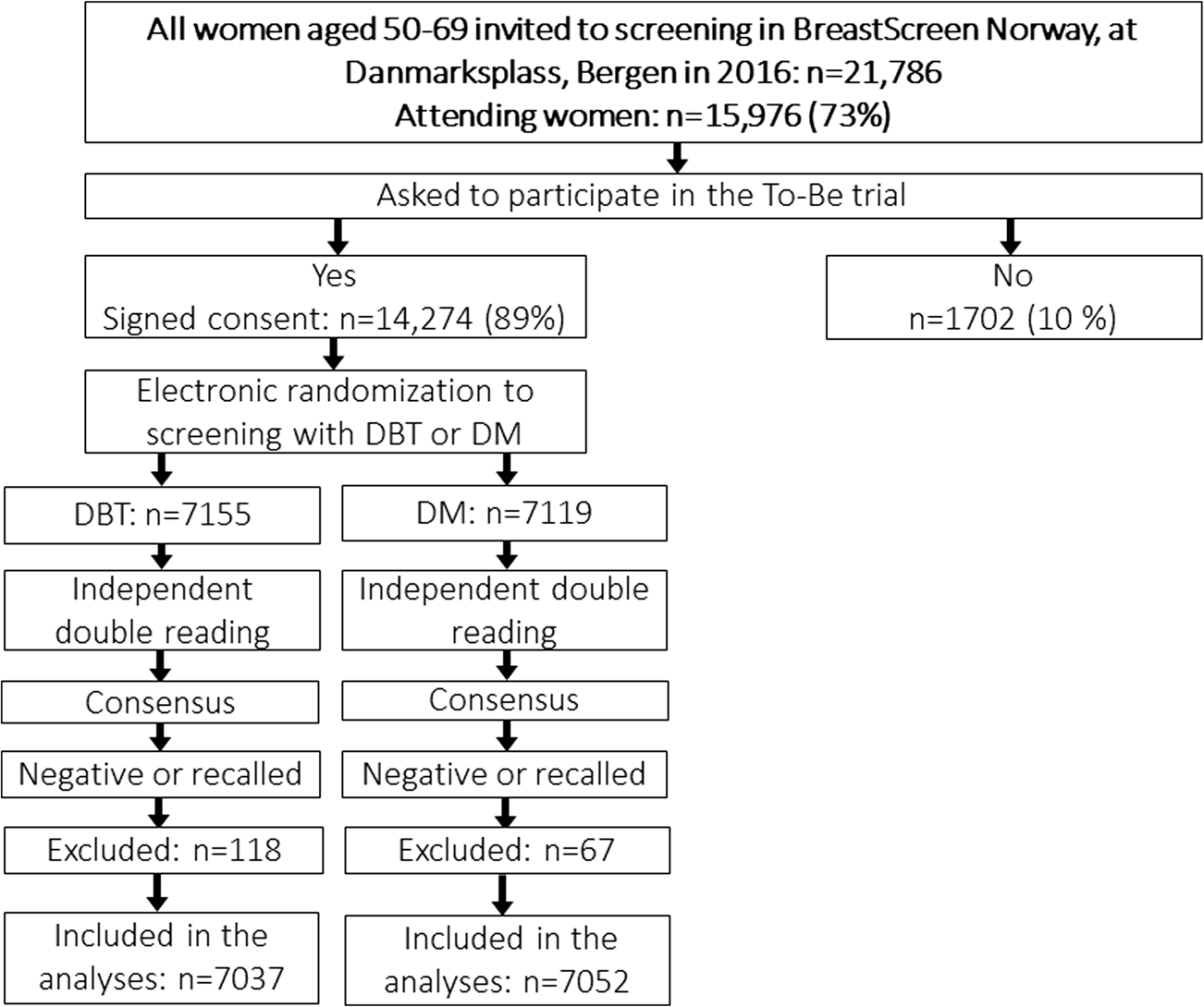
Study design and study population for interim analyses after 1 year of running the To-Be trial in Bergen, 2016

These first results from the To-Be trial reports pre-planned interim analyses of selected secondary outcome measures from the first year of To-Be, 2016. A total of 21,786 women were invited to screening in Bergen, whereas 15,976 (73%) attended and 14,274 (89%) agreed to participate in the trial. Altogether, 7155 women were randomized to DBT and 7119 to DM (Fig. 2).

### Definition of secondary outcome measures

Examination time was measured as the time spent from when the woman entered the examination room until she left: time was manually registered using a stopwatch for 438 and 535 randomly selected women screened with DBT and DM, respectively, during March 2017.

Time spent on initial screen reading and consensus was measured from the time the radiologist entered the women’s ID on the computer until the result of the reading/consensus was registered, using software developed for the trial. Initial screen reading time was measured for each radiologist, while consensus time was measured per woman without taking the number of participating radiologists into account.

The consensus rate was defined as the number of screening examinations discussed at consensus, divided by the total number of screening examinations. For each radiologist, the rate was estimated as the number of examinations given a score of 2 or higher (2+) divided by the number of screen readings per radiologist. The recall rate was the number of women recalled (post-consensus) because of abnormal mammographic findings divided by the number of women screened. For each radiologist, the recall rate was estimated as the number of cases they had read which were discussed at consensus and recalled divided by the number of screen readings by that radiologist.

Measures of volumetric breast density (VBD) and mean radiation dose per exposure (mean glandular dose, MGD) were calculated from the raw image data and data extracted from the DICOM header, using automated software (Volpara version 1.5.1, Volpara Health Technologies Ltd, Wellington, NZ) [18]. Average MGD per screening examination was calculated as the sum of the radiation doses reported by the software for both views and breasts divided by two. VBD was classified into a Volpara density grade (VDG) based on the following scale outlined by Volpara [19]: VDG 1 (VBD < 4.49%); VDG 2 (4.5–7.49%); VDG 3 (VBD 7.5–15.49%) and VDG 4 (VBD ≥ 15.5%). These categories are analogous to the BI-RADS 5th edition density categories a–d [20–22].

### Statistical analysis

We estimated mean and median time for screening examination, screen reading and consensus in minutes and seconds (minutes:seconds). For screen reading we excluded outlier values above 10 min and for consensus values above 15 min, assuming that radiologists had been interrupted. The outliers occurred similarly for DBT and DM. Further, we calculated mean values of MGD per examination. Rates of consensus and recall were presented per 100 screening examinations with 95% confidence intervals (95% CI). Analyses were stratified by screening technique (DBT and DM), screening history (prevalent or subsequent attendance), time since trial commencement (1–4, 5–8 and 9–12 months), the radiologists’ expertise in screen reading of DM before the start of the trial, and by cumulative number of DM and DBT screen reads in the trial, and mammographic density (VDG 1–4).

Trends in consensus and recall rates according to reading volume were tested for by a negative binomial regression model. We also used negative binomial regression to estimate the risk ratio (RR) and 95% confidence interval (95% CI) of consensus and recall for DBT using DM as the reference. Crude and adjusted RRs were calculated. Covariates in the adjusted models included mammographic density and an interaction effect between screening technique and density.

We used STATA version 15 (Stata Corp, TX) for all statistical analyses and tested differences across categories for statistical significance using two-sample *t*-tests, chi-square tests, ANOVA and tests of proportions (z test). A *p* value of less than 0.05 was considered statistically significant.

## Results

Among women included in the interim analyses, 1% (185/14,274) were excluded because of missing mammographic density data. Information from 14,089 women was thus included in analyses: 7037 screened with DBT and 7052 screened with DM. Women were, on average, 59 years old at screening in both groups (*p* = 0.469) (Table 1). The distribution of characteristics detailed in Table 1 did not differ between the two groups.

**Table 1:** Characteristics of the study population screened with digital breast tomosynthesis including synthesized 2D mammography (DBT) or digital mammography (DM) in the To-Be trial in Bergen, 2016

	DBT	DM	*p* value
	(*n* = 7037)	(*n* = 7052)	
Age (years)			
Mean/median	59/59	59/59	0.469*
50–54	27.6%	27.6%	0.983**
55–59	25.5%	25.8%	
60–64	24.9%	24.7%	
65–71	22.0%	21.9%	
Screening history (% of screened women)			0.883**
Prevalently screened	15.7%	15.6%	
Subsequently screened	84.4%	84.4%	
Mammographic density			0.248**
VDG 1	21.0%	20.4%	
VDG 2	44.8%	43.7%	
VDG 3	26.1%	27.1%	
VDG 4	8.2%	8.8%	

*t-test for means

**Chi-square test

Women spent an average time (minutes:seconds) of 5:24 (median 5:13) for DBT and 4:19 (median 4:07) for DM in the screening examination room (*p* < 0.01) (Table 2). Average and median times spent on initial screen reading and consensus were generally higher for DBT compared to DM.

**Table 2:** Mean and median time spent in the examination room per woman, at initial screen reading per radiologist, and at consensus for digital breast tomosynthesis with synthesized 2D (DBT) versus digital mammography (DM), in the To-Be trial in Bergen, 2016

	DBT	DM	*p* value*
Examination time per woman	*N* = 438	*N* = 534	
Mean/median (min:s)	5:24/5:13	4:19/4:07	< 0.01
Initial screen reading time per reader (min:s)	*N* = 7029	*N* = 7048	
All screens	1:11/0:54	0:41/0:26	< 0.01
Prevalent screens	1:10/0:53	0:33/0:19	< 0.01
Subsequent screens	1:11/0:54	0:43/0.27	< 0.01
*p* for trend*	0.850	< 0.01	
Reading time stratified by time since start of trial			
1–4 months	1:18/1:00	0:42/0:29	< 0.01
5–8 months	0:56/0.46	0:33/0:21	< 0.01
9–12 months	1:11/0.54	0:45/0:27	< 0.01
*p* for trend**	< 0.001	< 0.001	
Reading time stratified by mammographic density			
VDG 1	1:01/0:47	0:39/0:24	< 0.01
VDG 2	1:09/0:55	0:40/0:26	< 0.01
VDG 3	1:15/0:58	0:44/0:28	< 0.01
VDG 4	1:17/0:58	0:42/0:28	< 0.01
*p* for trend**	< 0.001	< 0.001	
Time spent on consensus (min:s)	*N* = 451	*N* = 519	
All	3:12/2:42	2:12/1:55	< 0.01
Prevalent screens	2:51/2:27	1:51/1:36	< 0.01
Subsequent screens	3:22/2:49	2:20/2:04	< 0.01
*p* for trend*	< 0.001	< 0.001	
Consensus time stratified by time since start of trial			
1–4 months	3:31/3:14	2:08/1:48	< 0.01
5–8 months	2:45/2:14	1:54/1:42	< 0.01
9–12 months	3:06/2:39	2:21/2:05	< 0.01
*p* for trend**	0.012	0.014	
Consensus time stratified by mammographic density			
VDG 1	3:15/2:33	2:15/2:03	< 0.01
VDG 2	3:14/2:47	2:12/1:51	< 0.01
VDG 3	3:16/2:48	2:14/1:56	< 0.01
VDG 4	2:52/2:30	2:00/1:51	< 0.01
*p* for trend**	0.623	0.695	

*t-test for means

**ANOVA

The rates of cases discussed at consensus were 6.4% for DBT and 7.4% for DM (*p* = 0.03) (Table 3). These rates did not differ among prevalent examinations (13.0% for both DBT and DM, *p* = 0.97), which was in contrast to the subsequent examinations, where the rate was 5.2% for DBT and 6.3% for DM (*p* < 0.01). We observed an increasing rate of cases discussed at consensus by VDG for DBT (*p* for trend < 0.01), but not for DM (*p* for trend = 0.078).

**Table 3:** Numbers (*n*) and percentages (%) of screening examinations discussed at consensus and recalls for digital breast tomosynthesis with synthesized 2D (DBT) versus digital mammography (DM), in the To-Be trial in Bergen, 2016

	Discussed at consensus			Recalled		
	DBT (*n* = 7037)	DM (*n* = 7052)	*p* value**	DBT (*n* = 7037)	DM (*n* = 7052)	*p* value**
	*N* % (95% CI)	*N* % (95% CI)		*N* % (95% CI)	*N* % (95% CI)	
All screens	451/7037 6.4% (5.8–7.0)	519/7052 7.4% (6.8–8.0)	0.03	208/7037 3.0% (2.6–3.4)	254/7052 3.6% (3.2–4.0)	0.03
Prevalent screens	143/1101 13.0% (11.0–15.0)	143/1097 13.0 (11.0–15.0)	0.97	69/1101 6.3% (4.8–7.7)	68/1097 6.2% (4.8–7.6)	0.95
Subsequent screens	308/5936 5.2% (4.6–5.8)	376/5955 6.3% (5.7–6.9)	< 0.01	139/5936 2.3% (2.0–2.7)	186/5955 3.1% (2.7–3.6)	< 0.01
*p* for trend*	< 0.01	< 0.01		< 0.01	< 0.01	
Time since start of trial						
1–4 months	175/2676 6.5% (5.6–7.5)	190/2641 7.2% (6.2–8.2)	0.35	81/2676 3.0% (2.4–3.7)	95/2641 3.6% (2.9–4.3)	0.25
5–8 months	76/1431 5.3% (4.2–6.5)	83/1463 5.7% (4.5–6.9)	0.67	37/1431 2.6% (1.8–3.4)	29/1463 2.0% (1.3–2.7)	0.28
9–12 months	200/2930 6.8% (5.9–7.7)	246/2948 8.3% (7.3–9.3)	0.03	90/2930 3.1% (2.4–3.7)	130/2948 4.4% (3.7–5.2)	< 0.01
*p* for trend*	0.149	< 0.01		0.648	< 0.01	
Mammographic density						
VDG 1	63/1475 4.3% (3.2–5.3)	87/1441 6.0% (4.8–7.3)	0.03	32/1475 2.2% (1.4–2.9)	49/1441 3.4% (2.5–4.3)	0.04
VDG 2	189/3150 6.0% (5.2–6.8)	224/3082 7.3% (6.4–8.2)	0.04	78/3150 2.5% (1.9–3.0)	110/3082 3.6% (2.9–4.2)	0.01
VDG 3	148/1836 8.1% (6.8–9.3)	154/1910 8.1% (6.8–9.3)	1.0	77/1836 4.2% (3.3–5.1)	73/1910 3.8% (3.0–4.7)	0.56
VDG 4	51/576 8.9% (6.5–11.2)	54/619 8.7% (6.5–10.9)	0.94	21/576 3.6% (2.1–5.2)	22/619 3.6% (2.1–5.0)	0.93
*p* for trend*	< 0.01	0.078		< 0.01	0.93	

*t-test for means

**ANOVA

The eight radiologists’ reading volume before and during the trial period varied (Appendix, Table 5). A score of 2+, resulting in a consensus meeting, was given for an average of 4.5% of the DBT and 5.4% of the DM screen reads for each of the radiologists (Appendix, Table 6). The consensus rate decreased with 0.1% for DBT (*p* = 0.4) and 0.2% for DM (*p* = 0.05) per 1000 DM screen reads prior to start-up of the trial.

The recall rate was 3.0% for DBT and 3.6% for DM (*p* = 0.03) (Table 3). This rate did not differ for the two techniques among prevalently screened women (6.3% for DBT and 6.2% for DM, *p* = 0.95), in contrast to subsequently screened women where the rate was 2.3% for DBT and 3.1% for DM (*p* < 0.01). For DBT, recall rates increased from 2.2% for women with VDG 1 to 3.6% for women with VDG 4 (*p* for trend < 0.01). No statistically significant difference was observed for women screened with DM (*p* = 0.93). The number of DM screen reads before the trial period did not significantly alter the recall rates for DBT or DM (*p* = 0.6 for DBT and *p* = 0.8 for DM) (Appendix Table 5).

The cumulative reading volume of DBT during the trial showed a non-significant trend of a decreasing consensus rate (RR = 0.95, *p* = 0.3) (Appendix Table 6 and Fig. 4). For DM, this trend reached statistical significance (RR = 0.93, *p* = 0.04). For recall rates, a non-significant trend of decreasing value with cumulative reading volume during the trial period was observed both for DBT and DM (*p* = 0.8 for DBT and *p* = 0.4 for DM).

The adjusted risks of consensus and recall were lower for DBT than for DM: RR 0.71 (95% CI 0.52–0.97) for consensus and 0.58 (95% CI 0.38–0.89) for recalls (Table 4). The interaction between screening technique and mammographic density was not stastistically significant when modelling the risk of consensus. However, the risk of recall among women screened with DBT increased for VDG 3 versus VDG 1 (*p* = 0.033), and displayed a trend toward increased values for VDG 4 versus VDG 1 (*p* = 0.061), compared with DM.

**Table 4:** Risk ratio (RR) of undergoing consensus and being recalled adjusted for mammographic density for digital breast tomosynthesis with synthesized 2D (DBT) versus digital mammography (DM) in the To-Be trial in Bergen, 2016

	RR of consensus			RR of recall		
	RR	95% CI	*p* value	RR	95% CI	*p* value
Screening technique						
DM	1.00	–	–	1.00	–	–
DBT	0.71	(0.52–0.97)	0.032	0.58	(0.38–0.89)	0.013
Mammographic density						
VDG 1	1.00	–	–	1.00	–	–
VDG 2	1.20	(0.95–1.53)	0.129	1.00	(0.73–1.37)	0.979
VDG 3	1.34	(1.04–1.72)	0.025	1.14	(0.81–1.59)	0.472
VDG 4	1.44	(1.04–2.00)	0.027	1.08	(0.68–1.72)	0.752
Screening technique and mammographic density (interaction)						
DBT–VDG 1	1.00	–	–	1.00	–	–
DBT–VDG 2	1.17	(0.81–1.68)	0.410	1.31	(0.79–2.18)	0.302
DBT–VDG 3	1.41	(0.96–2.07)	0.077	1.77	(1.05–3.01)	0.033
DBT–VDG 4	1.43	(0.88–2.33)	0.143	1.93	(0.97–3.84)	0.061

**Fig. 3:**
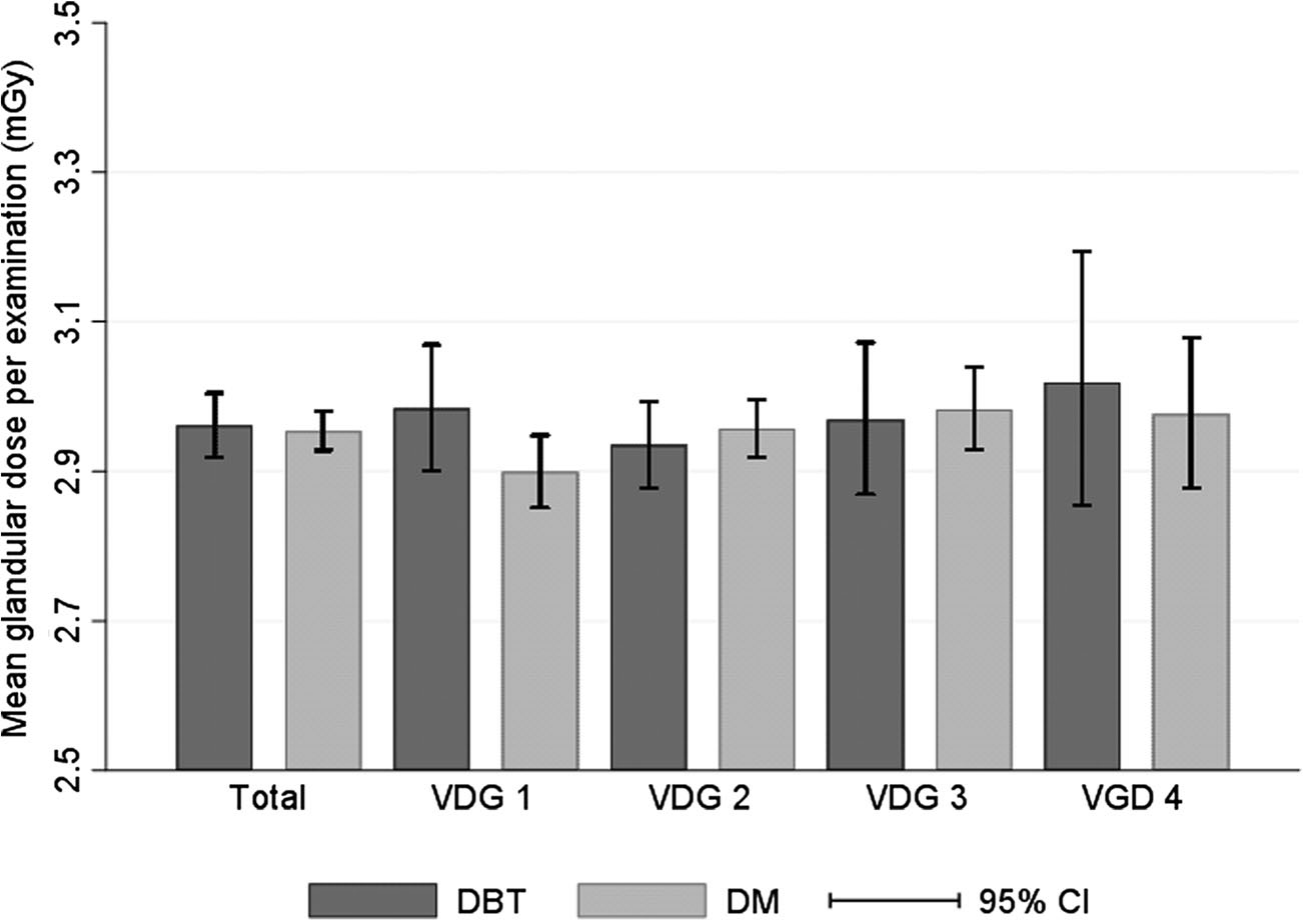
Mean glandular dose (MGD) per examination among women screened during the first year of the To-Be trial, overall and by Volpara density grade (VDG), stratified by imaging technique (digital breast tomosynthesis including synthesized 2D mammograms [DBT] or digital mammography [DM])

**Fig. 4:**
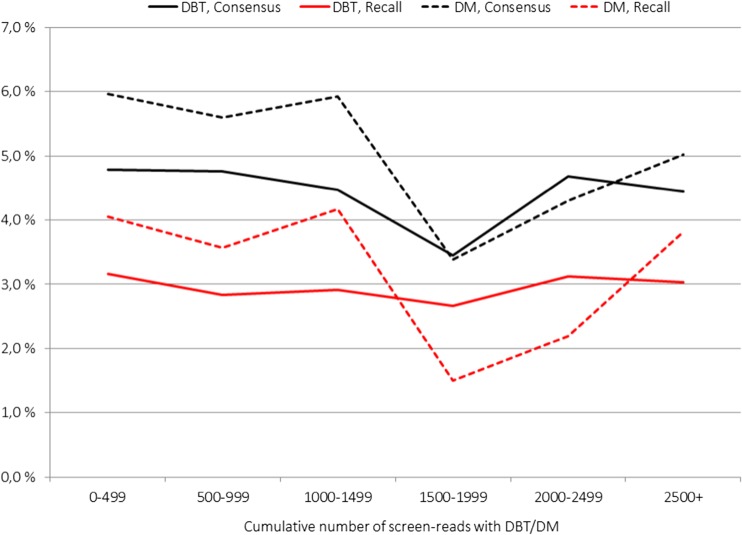
Rates of consensus and recalls by cumulative number of DM (dotted line) and DBT (solid line) screen-readings during the first year of the To-Be trial in Bergen, 2016

MGD per examination was 2.96 mGy for DBT and 2.95 mGy for DM (*p* = 0.433) (Fig. 3). It did not differ with mammographic density, nor within the density groups or between screening techniques.

## Discussion

In the first year of this RCT using DBT and DM in population-based breast cancer screening, we found lower consensus and recall rates among women screened with DBT than with DM. Our density-stratified analyses identified that recall rates were lower for DBT only for women with non-dense breasts (VDG 1 and VDG 2). Time spent both on screen reading and consensus was longer for DBT than for DM. Average MGD did not differ between the two techniques.

The lower recall rate for DBT compared to DM found in our interim analyses supports results from other studies, although recall rates have been shown to vary [1–4, 8, 9]. Different reading protocols and screening logistics might be some of the reasons for this variance [23–26]. Reducing recall rates below 3% in organized screening programs seems more challenging than reducing a recall rate of 10% or higher. Regardless of screening technique, there is limited evidence on what the ideal recall rate is, according to false positive screening results, cancer detection and breast cancer mortality [27, 28].

More than 65% of the women in our study were classified as having non-dense breast (VDG 1 or VDG 2). Women with non-dense breasts had a lower recall rate when screened with DBT than when screened with DM. However, recall rates did not differ between DBT and DM for women with dense breasts (VDG 3 or VDG4). Moreover, the effect of mammographic density on the risk of recall tended to be larger for DBT than for DM, a relevant finding in a breast cancer screening program given that it applies to the larger proportion of screening attendees in our population. Given the established knowledge about the increasing risk of breast cancer with mammographic density, the increase in recall rate with density seems reasonable.

The consenus rates were also higher for women with dense rather than fatty breasts, both for DBT and DM. This is possibly related to the complex parenchyma and the need for a second opinion. The consensus meeting used in BreastScreen Norway can be considered an educational activity where “positive” cases are discussed and prior screening exams are carefully considered before a final decision about recall is made. In a broader perspective, our results, demonstrating a lower percentage of cases needing to be discussed at consensus, suggest that DBT may reduce the percentage of cases needing third arbitrating reads in other programs. As far as we know, no other studies have reported consensus rates for DBT previously. It is possible that the dense cases discussed at consensus were more obvious to recall than the fatty cases. The radiologists might thus need less time to agree about recall for the dense versus the fatty cases.

The burden of increased examination and screen reading time from DBT is a critical issue for screening programs. The increased examination time was mainly due to time spent on explaining to the women how the x-ray machine would move and to make the x-ray tube ready for exposure. This extra time is expected to be reduced or resolved in subsequent screening rounds. We demonstrated that the average reading time was 30 s longer for DBT than for DM at initial screen reading (1:11 versus 0:41, respectively). The Oslo Tomosynthesis Screening Trial (OTST) reported that an additional 41 s was needed for reading DBT compared to DM [2], while results from the STORM trial, Malmo trial and a study by Dang et al showed an increase of 44 s [1], 30 s [4] and 54 s [29], respectively. Our results therefore represent the minimum increase in time spent on initial screen reading reported in the literature to date. However, the reading time varied between radiologists. We found that some radiologists were fast readers while other used more time. We consider this variability amongst the radiologists as individual-related rather than trial-related since the findings were independent of screening technique and volume of screen reads during their career.

In our study, time spent on screen reading and consensus was lowest 5–8 months after the start of the trial. This could be because this period was during the summer months, when fewer women were screened, resulting in low power in the estimate. The low reading and consensus time could also be related to a learning effect. A workshop reviewing cancer cases dismissed by one of the two readers was performed 7–8 months after the start of the trial, as a part of the usual quality assurance in the program. This might have contributed to readers deliberating longer at screen reading and may account for the increased reading time in the third period, 8–12 months after trial commencement.

Results from other studies indicate the need for training and workshops before reading DBT in screening [30, 31]. In our study, the radiologists’ experience in DM screen reading before the trial period varied from beginners to very experienced, the latter with more than 100,000 screen reads during their career as a breast radiologist. Not all radiologists participated in screen reading DBT in the pilot, which was performed 8 weeks before the trial commenced. We identified a significant decreasing trend of consensus with reading volume during the trial for DM, but not for DBT. The volume of screen reads prior to the trial did not show any correlation with either consensus or recall rate, neither for DBT nor DM. Our study presents results only for the first year of the trial, which might be considered the learning period. Further analyses including a longer study period might shed a different light on the issue. In this trial radiologists without experience in screen reading did training on test sets, shadow reading within the trial and performed clinical mammography with DBT. In retrospect, the pilot could have been extended to 6 months to enhance reader preparation, and additional workshops could have been held to make sure all participating radiologists had read a minimum number of negative and false positive examinations, screen-detected and interval breast cancers before the trial started. Although a roster was established at the start of the trial to ensure all radiologists read equal numbers of DBT and DM cases, this plan was not strictly followed because of varying individual work speeds and an unforeseen high volume of mammography outside of the screening program. Moreover, participating radiologists were not all exposed to the same number of DBT cases. The issues encountered in the implementation of the To-Be trial represent real-world screening challenges and provide novel insights that should inform other breast screening programs when planning DBT evaluations.

We found no statistically significant difference in radiation dose per examination between DBT and DM. Gennaro et al [10] reported doses per view (CC, MLO), also calculated by Volpara, for examinations acquired using a different unit/system and found the doses to be statistically significantly higher for DBT than for DM for both views. In a per view comparison (DBT and DM exposures of the same breasts during the same compression session) they found an average increase in DBT dose compared to DM of 38% (range 0–75%). Similarly, the Oslo Tomosynthesis Screening Trial used DBT systems from the same vendor as Gennaro et al and found, on average, dose per view to be 23% higher with DBT than DM when machine-reported doses were compared [12].

Using a system from yet another manufacturer, Lång et al [4] did not report dose values; instead, the automatic exposure control was set to yield an average dose of 1.2 mGy for DM and 1.6 mGy for DBT for a standard breast model. This gives an expected per view ratio of MGD_DBT_/MGD_DM_ of 1.33. For our system the manufacturer stated that the target MGD for DBT using automatic exposure control was equivalent to the MGD per view for DM, i.e. an expected ratio of approximately 1. The absence of a difference between MGD with DBT and DM observed in our study is therefore in line with how the system is set to operate by the manufacturer.

During the study period, routine quality assurance of the collected data and control activities were performed. We consider this to be one important strength of this study. We used an RCT design, the most reliable research design to compare screening modalities, and embedded this in a population-based screening program; these features of our trial minimize bias and increase the generalizability to other organized screening programs.

A limitation of this study is the short time spent on training and workshops in DBT for radiographers before the start of the trial, which could have influenced the results in either direction [30, 31]. Moreover, we have not presented breast cancer detection data; this decision was based on per protocol power estimation, which showed that 2 years of screening—one screening round—was needed to show a 25–30% difference in the rate of screen-detected breast cancer between DBT and DM. The moderate number of cases included in the analyses also represents a limitation in this study, particularly when stratifying into subgroups. Despite these limitations, we present our interim results to inform other population-based screening programs of selected secondary screening outcomes from an RCT of DBT and DM, in particular the estimated recall rate, screen reading time and radiation metrics, all of which matter to screening practice and research planning. To the best of our knowledge, there are no published secondary screening outcomes from other RCTs of DBT screening.

In conclusion, after the first year of running an RCT comparing DBT and DM, including about 7000 screened women in each arm, we showed a lower recall rate for women screened with DBT than DM. Our RCT sheds further light on the burdens of interpretation time and radiation dose, which are key factors in population-based screening. Time spent on screen reading and on consensus was longer for DBT than for DM. MGD measured by automated software on a GE SenoClair machine did not differ between the two techniques. Our results are somewhat different from other published studies and call for RCTs from different screening populations and with equipment from different vendors in order to gain evidence about the consequences of implementing DBT with synthesized mammograms, as a screening technique in population-based screening programs.

## Appendix

**Table 5:** Characteristics of the radiologists involved in the To-Be trial in Bergen, screen-reads (n), rates of consensus (score 2+) and recalls for DBT and DM by radiologist

Radiologist	Age	Started screen-reading in BreastScreen Norway (month, year)	Started screen-reading DBT in the trial (month, year)	DM screen-readings before the trial period (n)	Screen reads (n)		Score 2+ Consensus (%)		Recall (%)	
					DBT	DM	DBT	DM	DBT	DM
R1	36	Feb 2016	Feb 2016	0	2978	3884	4.1 %	5.3 %	2.4 %	3.1 %
R2	32	May 2014	Apr 2016	10744	920	383	7.1 %	8.1 %	3.8 %	3.7 %
R3	47	Oct 2010	Jan 2016	15085	1781	1344	4.3 %	4.9 %	3.1 %	3.8 %
R4	36	May 2012	Jan 2016	23801	1208	1563	4.4 %	6.7 %	3.1 %	4.7 %
R5	50	Jan 2009	Aug 2016	24015	453	502	4.6 %	9.2 %	3.1 %	4.4 %
R6	43	Sept 2007	Jan 2016	37361	1634	1177	4.8 %	5.2 %	3.1 %	3.8 %
R7	40	Sept 2008	Jan 2016	92590	2155	3789	4.2 %	4.0 %	2.7 %	3.0 %
R8	50	Sept 1997	Jan 2016	109152	2945	1462	4.4 %	6.6 %	3.2 %	4.9 %
*p for trend*							*0.4*	*0.05*	*0.6*	*0.8*
Total					14 074	14 104	4.5 %	5.4 %	3.0 %	3.6 %

*p* for trend tested by a negative binomial regression model

**Table 6:** Cumulative number of DBT and DM screen-reads during the first year of the To-Be trial in Bergen and subsequent rates of consensus and recalls

Number of DBT screen-reads in To-Be before current reading	Screening examinations (n)	Consensus (%)	Recall (%)
0-499	3955	4.8 %	3.2 %
500-999	3420	4.8 %	2.8 %
1000-1499	2707	4.5 %	2.9 %
1500-1999	1914	3.4 %	2.7 %
2000-2499	1155	4.7 %	3.1 %
2500+	923	4.4 %	3.0 %
*p for trend*		*0.3*	*0.8*
Total DBT	14074	4.5 %	3.0 %
Number of DM screen-reads in To-Be before current reading			
0-499	3922	6,0 %	4,1 %
500-999	3002	5,6 %	3,6 %
1000-1499	2446	5,9 %	4,2 %
1500-1999	1063	3,4 %	1,5 %
2000-2499	1000	4,3 %	2,2 %
2500+	2671	5,0 %	3,8 %
*p for trend*		*0.04*	*0.4*
Total DM	14104	5,4 %	3,6 %

*p* for trend tested by a negative binomial regression model

## Abbreviations

CC: Craniocaudal
DBT: Digital breast thomosynthesis
DM: Digital mammography
MGD: Mean glandular dose
MLO: Mediolateral oblique
RCT: Randomized controlled trial
SD: Standard deviation
SM: Synthetic two-dimensional mammogram
VBD: Volumetric breast density
VDG: Volpara density grade
